# Supplementary material for: Guided accumulation of active particles by topological design of a second-order skin effect
Source: Nat Commun. 2021 Aug 3;12:4691. doi: 10.1038/s41467-021-24948-2 (PMC8333048; doi:10.1038/s41467-021-24948-2)
Supplement: Supplementary file 1 — Supplementary Information: Guided accumulation of active particles by topological design of a second-order skin effect [file 41467_2021_24948_MOESM1_ESM.pdf]

Supplementary Information: Guided accumulation of active particles by topological design of a second-order skin effect

Lucas S. Palacios,[1] Serguei Tchoumakov,[2] Maria Guix,[1] Ignacio Pagonabarraga,[3,4,5] Samuel Sánchez,[1,6] and Adolfo G. Grushin[2]

[1]*Institute for Bioengineering of Catalonia (IBEC), Barcelona Institute for Science and Technology (BIST), Baldiri I Reixac 10-12, 08028 Barcelona, Spain*
[2]*Univ. Grenoble Alpes, CNRS, Grenoble INP, Institut Néel, 38000 Grenoble, France*
[3]*Departament de Física de la Matèria Condensada, Universitat de Barcelona, C. Martí Franquès 1, 08028 Barcelona, Spain*
[4]*University of Barcelona Institute of Complex Systems (UBICS), Universitat de Barcelona, 08028 Barcelona, Spain*
[5]*CECAM, Centre Européen de Calcul Atomique et Moléculaire, École Polytechnique Fédérale de Lausanne (EPFL), Batochime, Avenue Forel 2, 1015 Lausanne, Switzerland*
[6]*Institució Catalana de Recerca i Estudis Avançats (ICREA), Pg. Lluís Companys 23, 08010 Barcelona, Spain.*

(Dated: June 7, 2021)

SUPPLEMENTARY FIGURES

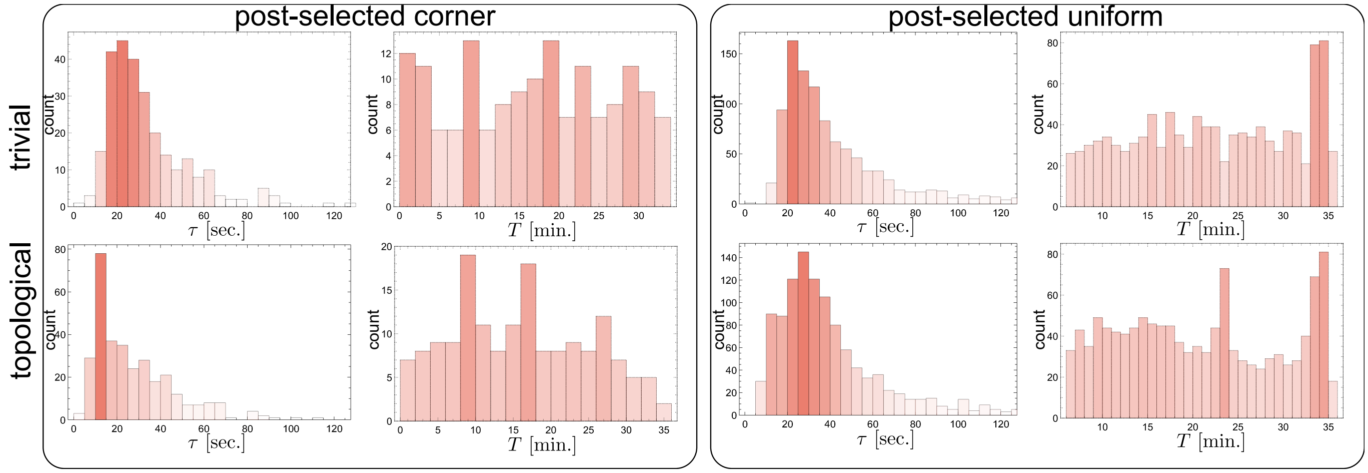

Supplementary Figure 1. Distribution of characteristic times for each selection of particles for the devices with $(L_x, L_y) = (13, 14)$ and a small density of active particles, for both topological and trivial devices. The timescale τ corresponds to the average time for a particle to go from one cell to the next. The timescale T corresponds to the total length of a trajectory.

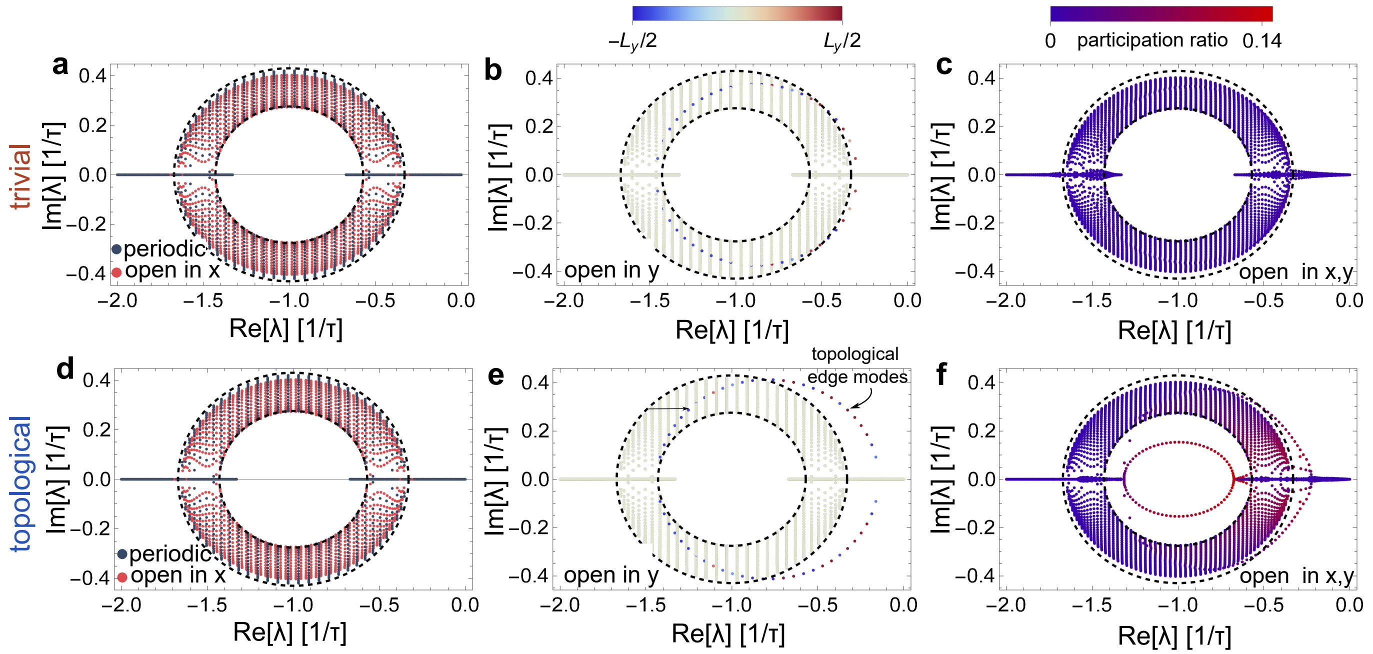

Supplementary Figure 2. Parametric representation of the real and imaginary part of the spectrum of the normal modes for the trivial **a**-**c** and topological **d**-**f** devices, for (L_x,L_y)=(L,L) = (70,70) lattice sites. **a,d** Spectrum for periodic boundary conditions in both directions (in blue) superimposed with that for open boundary conditions in x (in red). The two spectra coincide and indicate no edge mode. **b,e** Spectrum for open boundary conditions in y. The points are colored with respect to the average position $\langle y \rangle$ of the normal mode. The topological edge modes (26) partly hybridize with bulk modes, this is seen as a gap opening for larger values of $\mathrm{Re}[\lambda]$. **c,f** Spectrum for open boundary conditions in x and y. The points are colored with respect to the participation ratio $\sum_{\sigma ij} |P_{\sigma ij}|^4$ of the normal mode, a quantity which is small for delocalized modes. As shown in the main text, the modes in the inner dashed circle are localized at the top right and bottom left corners and are related to the second order non-Hermitian skin-effect.

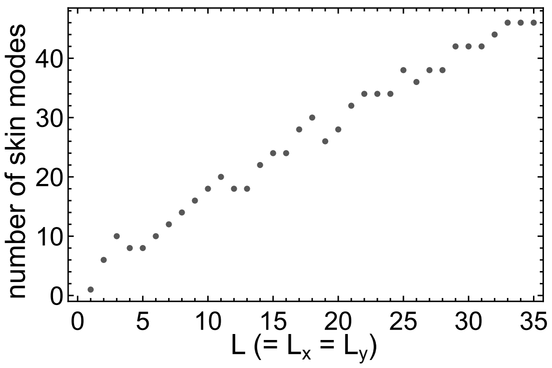

Supplementary Figure 3. Number of skin modes for open boundary conditions in both x and y directions for the topological device, as a function of system size L where $(L_x, L_y) = (L, L)$. This number corresponds to the number of states for open boundary conditions within the point gap found with periodic boundary conditions (i.e. within the inner circle in Supplementary Fig. 2f). The number of skin modes increases linearly with the perimeter, which is characteristic of a second-order non-Hermitian skin effect [6].

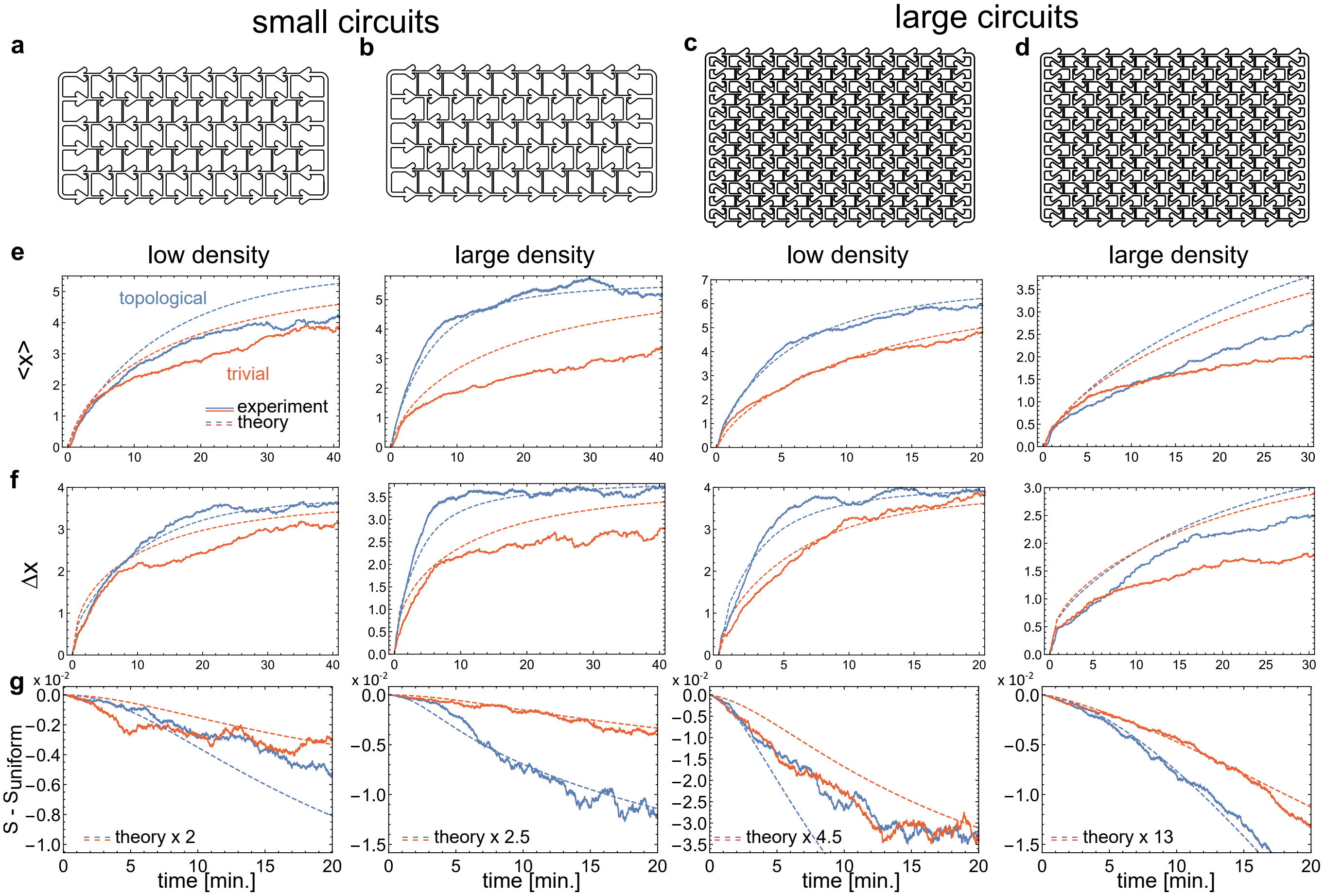

Supplementary Figure 4. **a**,**b**,**c**,**d** Depiction of the microfluidic devices used in our experiments. The trivial **a** and topological **b** small, $(L_x, L_y) = (12, 6)$, designs, and the trivial **c** and topological **d** large, $(L_x, L_y) = (13, 14)$, designs. In the main text we focus on the results obtained for the designs **c** and **d**. We consider four experimental situations with small $(L_x, L_y) = (12, 6)$ and large $(L_x, L_y) = (13, 14)$ microfluidic devices, and with low and large density of active particles. A larger density of particles enhances the Brownian motion, but also decreasest the velocity of particles and leads to more clusters. **e**,**f** Complementing Figs. 2g, h, we show the the time evolution of the average position and spread for all devices, when tracking particles initially at the top left corner (see Methods, post-selection of trajectories). We compare these quantities for the trivial and topological devices between experiments and theory. The model parameters are assigned separately for each case, using the experimental data (see Section A), and there are no fitting parameters. **g** Complementing Figs. 4 e, we show the time evolution of the entropy when tracking particles initially spread uniformly over the device (see Methods, post-selection of trajectories).

SUPPLEMENTARY DISCUSSION 1 : STOCHASTIC MODEL OF THE DEVICE

The design of the device where the Janus particles move in is inspired by the coupled-wire construction [1–3]. In this construction one-dimensional wires are coupled vertically to construct topological phases. Within each device we describe the motion of active particles as a random walk described by the continuous-time Markov master equations

$$\begin{cases} \tau \frac{dP_{A,ij}}{dt} = t_1(P_{B,i,j+1} - P_{A,ij}) + t_2(P_{B,ij} - P_{A,ij}) + t_+ P_{A,i-1,j} - t_- P_{A,ij} + t_- P_{A,i+1,j} - t_+ P_{A,ij}, \\ \tau \frac{dP_{B,ij}}{dt} = t_1(P_{A,i,j-1} - P_{B,ij}) + t_2(P_{A,ij} - P_{B,ij}) + t_+ P_{B,i+1,j} - t_- P_{B,ij} + t_- P_{B,i-1,j} - t_+ P_{B,ij}, \end{cases} \tag{1}$$

where the probability distribution, $P_{\sigma,ij}$, is decomposed over the discrete coordinates of the network, with $\sigma \in (A, B)$ the sub-cell indexes (see Figs. 2 a,b) and $(i, j) \in \mathbb{N}^2$ are the unit cell coordinates. This equation is balanced to ensure probability conservation, $\sum_{\sigma ij} P_{\sigma,ij} = 1$, and also, since the equation is irreducible, it has a unique stationary probability distribution with $dP_{\text{st.}}/dt = 0$ [4]. It is convenient to introduce the transition matrix $\hat{\boldsymbol{W}}$ such that

$$\tau \frac{d\mathbf{P}}{dt} = \hat{\boldsymbol{W}} \mathbf{P}, \tag{2}$$

where $(\mathbf{P})_{\sigma ij} = P_{\sigma,ij}$. Eq. (1) has the form of the Schrödinger equation with a non-Hermitian Hamiltonian.

A. Experimental values

We fix the values of the parameters in Eq. (1) by counting the number of times we see a particle moving along the four types of bulk links in the experiment. We have performed the experiment for two device sizes, $(L_x, L_y) = (12, 6)$ and $(L_x, L_y) = (13, 14)$ (see Figs. 4 a-d) and, for a low and a high density of active particles (see Methods, Experimental Setup).

For each situation we obtain the following transition probabilities

1. For our largest device, with $(L_x, L_y) = (13, 14)$, and a low density of active particles:
   - $(t_1, t_2, t_+, t_-) = (0.154, 0.212, 0.512, 0.122)$ for the trivial device,
   - $(t_1, t_2, t_+, t_-) = (0.214, 0.128, 0.545, 0.112)$ for the topological device.

   These are the values we use to compare our experiment with the model in Figs. 3 c,d.
2. For our largest device, with $(L_x, L_y) = (13, 14)$, and a high density of active particles:
   - $(t_1, t_2, t_+, t_-) = (0.126, 0.206, 0.461, 0.207)$ for the trivial device,
   - $(t_1, t_2, t_+, t_-) = (0.203, 0.119, 0.466, 0.212)$ for the topological device.

   These are the values we use to compare our experiment with the model in Figs. 4 e.
3. For our smallest device, with $(L_x, L_y) = (12, 6)$, and a low density of active particles:
   - $(t_1, t_2, t_+, t_-) = (0.150, 0.199, 0.519, 0.132)$ for the trivial device,
   - $(t_1, t_2, t_+, t_-) = (0.179, 0.123, 0.579, 0.119)$ for the topological device.
4. For our smallest device, with $(L_x, L_y) = (12, 6)$, and a high density of active particles:
   - $(t_1, t_2, t_+, t_-) = (0.143, 0.191, 0.514, 0.152)$ for the trivial device,
   - $(t_1, t_2, t_+, t_-) = (0.215, 0.125, 0.541, 0.120)$ for the topological device.

We observe an asymmetry between vertical and horizontal motions since $t_1 + t_2 = 0.33 < 0.67 = t_+ + t_-$; the motion along the horizontal axis is easier because the ratchets are aligned while vertical micro-channels are not. This asymmetry in the design is a consequence of the spatial constraints during device fabrication and it also helps to distinguish better topological and trivial devices in the experiment. Indeed, the contribution of topological edge modes to the displacement of particles is largest for an asymmetric network with $t_1 + t_2 < t_+ + t_-$, because it increases the decay time $\tau_d = \tau/(t_1 + t_2)$ of the edge modes. This condition is satisfied in the present experiment because the ratchets favour the horizontal motion of the Janus particles. Also, one can expect to observe a ballistic regime after a time

$$\tau_b = (t_+ + t_-)\tau/(t_+ - t_-)^2, \tag{3}$$

after which $\langle x \rangle > \Delta x$. Since in our experiment $\tau_b/\tau_d = 1.3 > 1$, it allows us to observe the ballistic regime.

Another parameter that enters our model is the typical time, τ, for a particle to move from one cell to another. We evaluate this time separately for each active particle by dividing the total time of its trajectory by the number of times it goes from one cell to another. This time is different for each active particles for a variety of reasons, for example because of the differences in particle sizes or local chemical environment. We evaluate the probability distribution of τ, $\mathcal{P}(\tau)$, for each initial configuration we pick up (see Fig 1). When we compare our model with the experiment we average all the quantities over the probability distribution of τ. For example, for the average position we compute

$$\langle\langle x \rangle\rangle = \sum_{\tau} \langle x \rangle \mathcal{P}(\tau), \tag{4}$$

where the average position $\langle x \rangle = \sum_{\sigma ij} x_{\sigma ij} P_{\sigma ij}$ is evaluated with the probability distribution of our model, in Eq. (1), for a given value of τ. In the text we omit the double bracket notation but this averaging procedure is always performed.

B. Bulk solution

In the situation of a infinite or periodic lattice, we can decompose the solution over the basis of Bloch solutions such that $P_{\sigma ij} = \sum_{k_x,k_y} P_\sigma(\mathbf{k}) e^{i(k_x i + k_y j)}$ where $\{k_x, k_y\} = \{n\pi a/L, m\pi a/L\}$ with a the lattice spacing, L the lattice size and $\{n, m\} \in \mathbb{N}^2$. In this basis Eq. (1) can be written for each $\mathbf{k}$ independently, $\tau d\mathbf{P}_\mathbf{k}/dt = \hat{\boldsymbol{W}}_\mathbf{k} \mathbf{P}_\mathbf{k}$, with

$$\tau \frac{d}{dt} \begin{pmatrix} P_A(\mathbf{k}, t) \\ P_B(\mathbf{k}, t) \end{pmatrix} = \begin{pmatrix} -1 + t_+ e^{-ik_x} + t_- e^{ik_x} & t_1 e^{ik_y} + t_2 \\ t_1 e^{-ik_y} + t_2 & -1 + t_+ e^{ik_x} + t_- e^{-ik_x} \end{pmatrix} \begin{pmatrix} P_A(\mathbf{k}, t) \\ P_B(\mathbf{k}, t) \end{pmatrix}, \tag{5}$$

which we can diagonalize to write

$$\tau \partial_t P_\eta(\mathbf{k}, t) = \lambda_\eta(\mathbf{k}) P_\eta(\mathbf{k}, t), \tag{6}$$

where $\eta = \pm$ denotes the two eigensolutions with normal mode P_η and eigenvalue λ_η. The eigenvalues are

$$\lambda_\eta = -1 + (t_+ + t_-)\cos(k_x) \pm \sqrt{(t_2 + t_1 \cos(k_y))^2 + t_1^2 \sin^2(k_y) - (t_- - t_+)^2 \sin^2(k_x)}. \tag{7}$$

The time evolution of the normal modes is then $P_\eta(\mathbf{k}, t) = P_\eta(\mathbf{k}) e^{\lambda_{\eta\mathbf{k}} t}$, so if we define the distribution at $t = 0$ by

$$P(\mathbf{x}, t = 0) = \sum_{\mathbf{k}} \sum_{\eta} c_\eta(\mathbf{k}) P_\eta(\mathbf{k}) e^{i(k_x x + k_y y)}, \tag{8}$$

then the probability distribution at a time t is

$$P(\mathbf{x}, t) = \sum_{\mathbf{k}} \sum_{\eta} c_\eta(\mathbf{k}) P_\eta(\mathbf{k}) e^{\lambda_{\eta\mathbf{k}} t + i(k_x x + k_y y)}. \tag{9}$$

Since $\mathrm{Re}(\lambda_{\eta\mathbf{k}}) \leq 0$, with a maximum at $\mathbf{k} = 0$ with $\mathrm{Re}(\lambda_{\eta\mathbf{k}}) = 0$, the infinite or periodic lattice tends towards the uniform distribution in space at long times.

C. Real and imaginary eigenvalues

The imaginary and real parts of the eigenvalues λ_n determine the average position and variance of the active particles. Indeed, for an initial probability distribution that is uniform and that we decompose over the eigensolution, $P(\mathbf{x}, t = 0) = \sum_{\mathbf{k}} \sum_\eta c_\eta(\mathbf{k}) P_\eta(\mathbf{k}) e^{i(k_x x + k_y y)}$, we have

$$\langle r_i \rangle_t = \sum_{\mathbf{x}} r_i P(\mathbf{r}, t) = -\sum_{\eta=\pm} c_\eta(\mathbf{k} = 0) \partial_{k_i} \mathrm{Im}\left[\lambda_\eta(\mathbf{k} = 0)\right] t, \tag{10}$$

$$\Delta_t r_i^2 = \langle r_i^2 - \langle r_i \rangle_t^2 \rangle_t = -\sum_{\eta=\pm} c_\eta(\mathbf{k} = 0) \partial_{k_i}^2 \mathrm{Re}\left[\lambda_\eta(\mathbf{k} = 0)\right] t, \tag{11}$$

where $\mathbf{r} = (x, y)$ is the position over cells (see Fig. 1 e). These two relations show that for an initially uniform distribution, only the eigenvalues at $\mathbf{k} = 0$ matter. As explained in the main text, the slope of the imaginary part is related to the ballistic motion and the curvature of the real part is related to diffusion.

D. Edge modes

In this section we derive the spectrum of edge modes for open boundary conditions in the y direction, and periodic in x. Because the lattice is periodic in the x direction, we can still apply the procedure in Sec. B for the horizontal (x) direction. This leads to the equation

$$\tau\frac{d}{dt}\begin{pmatrix} P_{A,j}(k_x,t) \\ P_{B,j}(k_x,t)\end{pmatrix} = \begin{pmatrix} -1+t_+e^{-ik_x}+t_-e^{ik_x} & t_2 \\ t_2 & -1+t_+e^{ik_x}+t_-e^{-ik_x}\end{pmatrix}\begin{pmatrix} P_{A,j}(k_x,t) \\ P_{B,j}(k_x,t)\end{pmatrix} + t_1\begin{pmatrix} P_{B,j+1}(k_x,t) \\ P_{A,j-1}(k_x,t)\end{pmatrix}, \tag{12}$$

where j labels the lattice site in the vertical (y) direction. Since the bulk spectrum (7) is invariant by the transformation $k_y \to -k_y$, the solutions for open boundaries can be expanded as

$$P_{A,j} = a_+e^{ik_yj} + a_-e^{-ik_yj} = A_1\cos(k_yj) + A_2\sin(k_yj), \tag{13}$$

$$P_{B,j} = b_+e^{ik_yj} + b_-e^{-ik_yj} = B_1\cos(k_yj) + B_2\sin(k_yj). \tag{14}$$

The value of k_y is not necessarily real and is set by the boundary condition. Also, if we write $k_y = \chi + i\mu$, we have that

$$\cos(k_yj) = \cos(\xi j)\cosh(\mu j) - i\sin(\xi j)\sinh(\mu j), \tag{15}$$

$$\sin(k_yj) = \sin(\xi j)\cosh(\mu j) + i\cos(\xi j)\sinh(\mu j), \tag{16}$$

and when replacing these expressions in Eq. (7) we have to check that the real part of λ_n is negative, so the solutions stay normalized. We consider two types of open boundary conditions in y: (1) one that preserves chiral symmetry and, (2) one that preserves detailed balance.

As we show next, the boundary condition (1) is a useful approximation; its chiral symmetry allows us to show in a simple way why the edge states have a non-hermitian skin-effect in the next section. The price to pay is that we do not recover the exact numerical edge spectrum, but only an approximation to it. The boundary condition (2) is more involved analytically, but recovers the exact numerical edge spectrum with the same topological information, which is discussed in the next section.

(1) **Boundary condition with chiral symmetry.** A way to describe open boundary conditions is to solve Eq. (1) with the constraints $P_{B,L_y+1} = 0$ and $P_{A,0} = 0$. These constraints prevent a particle from moving away from the region $y \in [1, L_y]$. The constraint $P_{B,L_y+1} = 0$ implies that

$$P_{B,j} = B_1\frac{\sin(k_y(L_y+1-j))}{\sin(k_y(L_y+1))}. \tag{17}$$

This is input in the equation for $P_{A,0}$ in Eq. (12) to give the consistency relation

$$\frac{t_1}{t_2} = -\frac{\sin(k_y(L_y+1))}{\sin(k_yL_y)} \xrightarrow[Ly\to\infty]{k_y=\pi+i\mu} e^{\mu}. \tag{18}$$

This transcendental equation has L_y <u>real</u> solutions for $k_y \in [0,\pi)$ if only $t_2/t_1 < 1$, this corresponds to the trivial device which has no edge mode. If $t_1/t_2 > 1$, as in the topological device, this equation has only $L_y - 1$ real solutions for $k_y \in [0,\pi)$. The missing real solution is that at $k_y = \pi$. Note that it would be missing at $k_y = 0$ if $t_1/t_2 < -1$, but this does not occur since conditional probabilities are positive. This missing mode is actually substituted by an evanescent mode, with $k_y = \pi + i\mu$, solution to Eq. (18). In the limit where L_y is large, the first term in the square-root in Eq. (7) vanishes and the eigenvalues are

$$\lambda_{\chi=\pm} = -1 + (t_+ + t_-)\cos(k_x) + \chi i(t_- - t_+)\sin(k_x). \tag{19}$$

They correspond to the top ($\chi = +$) and bottom ($\chi = -$) interface, respectively. Compared to Eq.(19) the numerical edge modes seen in Supplementary Fig. 2e are shifted by a constant. To obtain the exact spectrum we discuss the second type of boundary conditions, that preserve detailed balance.

(2) **Boundary condition with detailed balance.** The previous boundary condition does not respect detailed balance because it removes the hopping term to outside the lattice without tuning-off the associated on-site sink. This on-site sink is the $-1 = -(t_1 + t_2 + t_+ + t_-)$ term in (5). Imposing detailed balance at the boundary leads to the following condition at the $j = L_y$ boundary,

$$\lambda P_{A,L_y} = (-1 + t_1 + t_+e^{-ik_x} + t_-e^{ik_x})P_{A,L_y} + t_2P_{B,L_y}. \tag{20}$$

This equation looks similar to the previous one, where we impose $P_{B,L_y+1} = 0$, but with an additional $t_1 P_{A,L_y}$ contribution. This boundary condition can be worked out with (1) and (7) to give

$$\left[2\cos(k_y) - \underbrace{\frac{t_2}{\lambda - (-1 + t_1 + t_+ e^{-ik_x} + t_- e^{ik_x})}}_{\equiv z} \right] P_{B,L_y} = P_{B,L_y-1}. \tag{21}$$

The second term in the bracket would be zero in the boundary condition (1). We combine (21) with Eq (13) and get

$$P_{B,j} = B_1 \left(\cos(kj) - \frac{\cos(k(L_y+1)) - z\cos(kL_y)}{\sin(k(L_y+1)) - z\sin(kL_y)} \sin(kj) \right). \tag{22}$$

We can then insert this in the equation for the $j = 1$ boundary, leading to

$$\lambda P_{B,1} = (-1 + t_1 + t_+ e^{ik_x} + t_- e^{-ik_x}) P_{B,1} + t_2 P_{A,1}, \tag{23}$$

and that can be transformed to

$$\frac{1}{z} = 2\cos(k_y) - \frac{P_{B,2}}{P_{B1}} = \frac{\sin(k_y(L_y+1)) - z\sin(kL_y)}{\sin(k_y L_y) - z\sin(k(L_y-1)} \xrightarrow[Ly\to\infty]{k_y=\pi+i\mu} -e^{\mu}. \tag{24}$$

This transcendental equation is similar to Eq. (18) and depending on the value of z, now also a function of λ and k_x, we loose a real-valued solution for k_y at $k_y \approx \pi$. This solution is replaced by an evanescent solution with $k_y = \pi + i\mu$ and in the limit of $L_y \to \infty$, we obtain

$$e^{\mu} = -1/z = -\frac{\lambda - (-1 + t_1 + t_+ e^{-ik_x} + t_- e^{ik_x})}{t_2}. \tag{25}$$

Then replacing $\cos(k) \to_{k\to\pi+i\mu} - \cosh(\mu) = \frac{1}{2}(z + 1/z)$ in the equation for λ (7), we find the two solutions

$$\lambda_{\chi=\pm} = h_0 + t_1 + \chi\sqrt{h_z^2 + t_2^2}, \tag{26}$$

where $h_0(k_x) = -1 + (t_+ + t_-)\cos(k_x)$ and $h_z(k_x) = i(t_- - t_+)\sin(k_x)$. These solutions coincide with the numerical spectrum in Supplementary Fig. 2e. The edge mode dispersion, $\lambda_\pm$ is close to that in Eq. (19) but with a spectrum shifted by t_1. For some values of k_x the spectrum of edge modes (26) hybridize with bulk modes. Compared to the boundary condition (1), this can be traced back to imposing detailed balance, which adds the t_1 term in (20), the sink potential.

E. Topological properties

The probability distribution of active particles is determined by the non-Hermitian matrix $\hat{\boldsymbol{W}}_{\mathbf{k}}$ in Eq. (5). Non-Hermitian operators are classified according to their symmetries, to the gap structure of their complex energy spectra and to their dimension [5]. Depending on the discrete symmetries of the system, and excluding crystal symmetries, the model falls in one of the 38 distinct topological classes that define strong topological insulators. The gaps are or three types: real or imaginary line gaps, or point gaps. If the class is topologically non-trivial for the given gap type and dimension, then a topological invariant exists to classify the possible topological phases of the system in that class. In our case, the matrix $\hat{\boldsymbol{W}}$ is non-Hermitian with time-reversal symmetry, $\hat{\boldsymbol{W}}_{\mathbf{k}}^* = \hat{\boldsymbol{W}}_{-\mathbf{k}}$, and inversion symmetry, $\hat{\sigma}_x \hat{\boldsymbol{W}}_{\mathbf{k}} \hat{\sigma}_x = \hat{\boldsymbol{W}}_{-\mathbf{k}}$. It falls in the real Altland-Zirnbauer (AZ) symmetry class AI. Since $\hat{\boldsymbol{W}}$ has a point gap (see Fig 2) and describes a two-dimensional system, this class is trivial and has no strong topological invariant [5].

Our model can instead be better understood by analogy to weak topological insulators. Weak topological insulators in d dimensions can be constructed by coupling $d-1$ strong topological insulators. Following this principle $\hat{\boldsymbol{W}}$ is constructed by coupling strong one-dimensional topological insulators defined in the y direction with non-reciprocal hoppings along the x direction. In this way $\hat{\boldsymbol{W}}$ in a direct sum of two terms $\hat{\boldsymbol{W}}_{\mathbf{k}} = \hat{\boldsymbol{W}}_x + \hat{\boldsymbol{W}}_y$ with

$$\hat{\boldsymbol{W}}_x = (-1 + (t_+ + t_-)\cos(k_x))\,\hat{\sigma}_0 + i(t_- - t_+)\sin(k_x)\hat{\sigma}_z, \tag{27}$$

$$\hat{\boldsymbol{W}}_y = (t_2 + t_1\cos(k_y))\,\hat{\sigma}_x - t_1\sin(k_y)\hat{\sigma}_y. \tag{28}$$

In these equations, $\hat{\boldsymbol{W}}_y$ describes a strong hermitian topological insulator in the y direction. It is the Su, Schrieffer and Heeger model [7]. $\hat{\boldsymbol{W}}_y$ has chiral symmetry, represented by $\hat{\sigma}_z$ satisfying $\{\hat{\boldsymbol{W}}_y, \hat{\sigma}_z\} = 0$, and it is time-reversal symmetric, represented by complex conjugation. Therefore, $\hat{\boldsymbol{W}}_y$ belongs to the real AZ symmetry class BDI. Since $\hat{\boldsymbol{W}}_y$ is one-dimensional and has a real line gap (it is Hermitian), it can be classified by a strong topological invariant [8], the total Berry phase on the Brillouin zone. It can be calculated for each sub-band $\eta = \pm$ in (6) as [9]

$$\gamma_{y\eta} = \frac{1}{2}\left(\gamma_{y\eta}^{LR} + \gamma_{y\eta}^{RL}\right) = \frac{1}{2\pi}\int_{-\pi}^{\pi} dk_y\, \partial_{k_y}\phi, \tag{29}$$

where we have introduced the Berry phase on the left (P_η^L) and right (P_η^R) normal modes of $\hat{\boldsymbol{W}}$

$$\gamma_{y\eta}^{\alpha\beta} = \frac{i}{2\pi}\oint_{\mathcal{C}} dk_y\, P_\eta^{\alpha *}(\mathbf{k})\partial_{k_y} P_\eta^{\beta}(\mathbf{k}). \tag{30}$$

In this last expression, the normal modes of $\hat{\boldsymbol{W}}$ are

$$P_\eta^\alpha = \frac{1}{\sqrt{1+e^{2i\alpha\theta}}}\begin{pmatrix} 1 \\ \eta e^{i(\phi+\alpha\theta)} \end{pmatrix}, \tag{31}$$

with $\alpha = +1$ for right- and $\alpha = -1$ for left-eigenstates, $\phi = \arg\left(t_2 + t_1\cos(k_y) + it_1\sin(k_y)\right)$, and $\theta = \arg\left(-\sum_i t_i + (t_+ + t_-)\cos(k_x) + i(t_- - t_+)\sin(k_x)\right)$. The topological phase occurs when $t_1 > t_2$ because $\gamma_{y\pm} = 1$, and the system is trivial otherwise, with $\gamma_{y\pm} = 0$. This winding number is independent on $\hat{\boldsymbol{W}}_x$ in (28), it is the same for both $\hat{\boldsymbol{W}}$ and $\hat{\boldsymbol{W}}_y$. Since the winding number $\gamma_{y\eta}$ defined in Eq. (29) is the winding number of the Hermitian operator $\hat{\boldsymbol{W}}_y$, we call it the Hermitian winding number, $w_{\mathrm{H}} = \gamma_y$.

The non-Hermitian matrix $\hat{\boldsymbol{W}}_x$ in Eq. (28) couples each copy of $\hat{\boldsymbol{W}}_y$ in the x direction. Because $\hat{\boldsymbol{W}}_x$ is time-reversal symmetric and has no chiral symmetry, it can be classified in the real AZ symmetry class AI. Also, since it describes a one-dimensional system with an imaginary line gap, it can be classified by a topological invariant. As we now show, both the winding of the spectrum in the complex plane and the total Berry phase vanish for $\hat{\boldsymbol{W}}_x$, which is thus always trivial [9]. The reason the winding numbers of $\hat{\boldsymbol{W}}_x$ vanish can be derived from the fact that $\hat{\boldsymbol{W}}_x$ is composed of two independent copies, one for each eigenvalue $\chi = \pm$ of $\hat{\sigma}_z$,

$$H_{s,\chi}(k_x) = -1 + (t_+ + t_-)\cos(k_x) + \chi i(t_- - t_+)\sin(k_x). \tag{32}$$

These two copies are non-Hermitian matrices with the same symmetries than $\hat{\boldsymbol{W}}_x$ and each with opposite winding numbers of their spectrum in the complex plane [4, 9, 10]

$$w_{\mathrm{nH},\pm} = \frac{1}{2\pi i}\int_{-\pi}^{\pi} dk_x\, \frac{d\log\left(H_{s,\pm}(k_x) - E\right)}{dk_x} = -w_{\mathrm{nH},\mp}. \tag{33}$$

These winding number are $w_{\mathrm{nH},\chi} = \chi(= \pm 1)$ for modes within an ellipse in the complex plane, centered at $\lambda_C = -1$ and with radii $t_+ + t_-$ on the real axis and $|t_+ - t_-|$ on the imaginary axis (the outer dashed black line in Fig. 4f). In general, these two winding numbers cancel out in $\hat{\boldsymbol{W}}$ since the two independent modes of $\hat{\boldsymbol{W}}_x$ are coupled by $\hat{\boldsymbol{W}}_y$ in the y direction. As a consequence, the spectrum for periodic and open boundary conditions in the x direction are similar to each other, so the non-Hermitian skin effect is absent (see Figs. 2 b,d).

As derived in Sec. D, in the topological device a y boundary that preserves chirality has two chiral edge modes, described by the two copies (32) on the top ($\chi = +$) and bottom ($\chi = -$) edges, since the chirality of $\hat{\boldsymbol{W}}_y$ is represemnted by $\hat{\sigma}_z$. In this case, each edge displays a one-dimensional first-order non-Hermitian skin effect because of their non-zero w_{nH}, resulting in a second-order non-Hermitian skin effect of the two-dimensional system.

Although the boundary condition that preserves chiral symmetry is a useful approximation, it is necessary to impose detailed balance at the top and bottom edge to obtain exactly the edge theory that we obtain numerically, as discussed in Sec. D. The main change between these two boundary conditions is that the spectrum of the edge modes shifts along the real axis, and as a result some of the edge modes partially hybridize with bulk modes (26), see Supplementary Fig. 2e. This effect can be interpreted as an edge potential on the topological edge states, which, despite breaking chiral symmetry, is not expected to change their skin-effect, for moderate parameter values. We confirm this expectation numerically, using the conditional probability parameters relevant for our experiments in Supplementary Fig. 2f.

With the above, our analysis implies that, if chiral edge modes exists with open boundary conditions in the y direction (i.e. when $t_1/t_2 > 1$), these display a non-Hermitian skin effect with open boundary conditions in the x direction, resulting in an

accumulation of active particles at the corners. This effect is a second-order non-Hermitian skin-effect [6, 11–14], which exists when the topological number

$$\nu = w_{\mathrm{H}} w_{\mathrm{nH}}, \tag{34}$$

is non-zero. This is the case for our topological devices, but $\nu = 0$ for our trivial devices. The Hermitian topology of $\hat{\boldsymbol{W}}_y$ when $w_{\mathrm{H}} \neq 0$ spatially separates the topological non-Hermitian modes of $\hat{\boldsymbol{W}}_x$ with opposite and non-vanishing w_{nH}, realizing a second-order skin effect.

F. Topological invariant of the second-order non-Hermitian skin-effect: connection to Hermitian topology

By following Ref. [13] it is possible to understand the second-order non-Hermitian skin effect by connecting with topological Hermitian systems as follows. First we construct an Hermitian matrix from $\hat{\boldsymbol{W}}$ as

$$H_{\mathrm{eff}} = \begin{pmatrix} 0 & \hat{\boldsymbol{W}} - \lambda_C \\ \hat{\boldsymbol{W}}^\dagger - \lambda_C & 0 \end{pmatrix}, \tag{35}$$

where λ_C is a base energy defined by the center of the point gap. This is in general a complex number, but, as can be seen from Supplementary Fig. 2, in our case $\lambda_C = -1$. When λ_C is real, as in our case, it is instructive to perform a unitary transformation of H_{eff} as $\tilde{H}_{\mathrm{eff}} = U H_{\mathrm{eff}} U^\dagger$ by using the unitary matrix

$$U = \begin{pmatrix} 0 & 0 & 0 & -1 \\ 1 & 0 & 0 & 0 \\ 0 & -1 & 0 & 0 \\ 0 & 0 & 1 & 0 \end{pmatrix}, \tag{36}$$

such that

$$\tilde{H}_{\mathrm{eff}} = \tilde{H}_x \otimes \tau_0 + \sigma_0 \otimes \tilde{H}_y \tag{37}$$

$$\tilde{H}_x = \left(-1 - \lambda_C + (t_+ + t_-)\cos(k_x)\right)\hat{\sigma}_x - (t_- - t_+)\sin(k_x)\hat{\sigma}_y, \tag{38}$$

$$\tilde{H}_y = \left(-t_2 - t_1\cos(k_y)\right)\hat{\tau}_x - t_1\sin(k_y)\hat{\tau}_y. \tag{39}$$

Both $\tilde{H}_x$ and $\tilde{H}_y$ have chiral symmetry represented by $\hat{\sigma}_z$ and $\hat{\tau}_z$, respectively. Each symmetry is associated to the winding numbers $\tilde{\omega}_{x,y}$. Consequently, $\tilde{H}_{\mathrm{eff}}$ has the symmetry $\tilde{\Gamma} = \hat{\sigma}_z \otimes \hat{\tau}_z$. Additionally it displays inversion symmetry given by $\tilde{I} = \hat{\sigma}_x \otimes \hat{\tau}_x$. Note that $\tilde{\Gamma}$ commutes with $\tilde{I}$.

As discussed in Sec E, the present model has trivial non-Hermitian topology in the bulk but non-trivial on the edges. In the nomenclature of Ref. [13] its second-order non-Hermitian skin effect is thus extrinsic, since chirality and inversion symmetries commute and the non-Hermitian topology is only characterized by chiral symmetry $\tilde{\Gamma}$. The corresponding Hermitian winding numbers of $\tilde{H}_x$ and $\tilde{H}_y$ are non-trivial and can be computed from (29). We have that

1. the normal modes of $\tilde{H}_x$ are

$$P_{\eta=\pm} = \frac{1}{\sqrt{2}} \begin{pmatrix} 1 \\ \eta e^{i\phi_x} \end{pmatrix} \tag{40}$$

   where $\phi_x = \arg\left[-(1+\lambda_C) + (t_+ + t_-)\cos(k_x) + i(t_+ - t_-)\sin(k_x)\right]$. So the winding number w_x is

$$w_x = \frac{1}{2\pi}\int_{-\pi}^{\pi} dk_x \partial_{k_x}\phi_x. \tag{41}$$

   $w_x = 1$ for $\lambda_C \in -1 \pm (t_+ + t_-)$ and it vanishes otherwise.

2. the normal modes of $\tilde{H}_y$ are

$$P_{\eta=\pm} = \frac{1}{\sqrt{2}} \begin{pmatrix} 1 \\ \eta e^{i\phi_y} \end{pmatrix} \tag{42}$$

where $\phi_y = \arg\left[-(t_2 + t_1\cos(k_y)) - it_1\sin(k_y)\right]$. So the winding number w_y is

$$w_y = \frac{1}{2\pi}\int_{-\pi}^{\pi} dk_y \partial_{k_x}\phi_y. \tag{43}$$

$w_y = 1$ for $t_1/t_2 > 1$, which defines the topological device, and it vanishes in the trivial device.

Comparing to our discussion in the previous section, we see here that the Hermitian winding number, w_{H}, of $\hat{\boldsymbol{W}}$ coincides with the total Berry phase w_y of $\tilde{H}_y$. Moreover, the non-Hermitian winding number, w_{nH}, coincides with the total Berry phase w_x of $\tilde{H}_x$ for eigenvalues λ_C on the real axis, as in our case. The topological invariant we introduced in the main text Eq. (3) (Eq. (34) in this Supplementary Material) thus coincides with the one proposed in Ref. [13]

$$\nu = w_{2D} \equiv w_x w_y, \tag{44}$$

for $\lambda_C \in \mathbb{R}$. Note that if we had considered $\lambda_C \in \mathbb{C}$, then there would be an additional chirality-breaking contribution $-\mathrm{Im}[\lambda_C]\hat{\sigma}_2 \otimes \hat{\tau}_3$ that enters (28), that would have prevented this topological classification [13].

G. Discussion on the topological invariant ν

In Sections E and F we computed the same topological invariant, ν, from two different arguments. One may wonder if this invariant is truly a 2D invariant. First, we note that ν is closer to invariants that characterize weak topological insulators, as it is defined by a combination of lower dimensional (in our case 1D) topological invariants. In this sense, the topological invariant describing our system is not a bulk 2D topological invariant. Rather, the present higher-order topological behaviour emerges from the superposition of Hermitian and non-Hermitian 1D topologies in transverse directions, that combined lead to a second-order non-Hermitian skin-effect.

We note as well that ν is not strictly a 1D invariant either since a first order non-Hermitian skin-effect cannot occur in the presence of inversion. As discussed in the main text, in our experiments we enforce detailed balance over the unit cell, so the lattice does not break inversion symmetry, and thus does not allow a first-order skin-effect. Each edge independently may break 1D inversion symmetry, and cause the particle accumulation. The parameters for which our 2D model allows this to happen are captured by ν.

SUPPLEMENTARY DISCUSSION 2 : COMPARISONS BETWEEN DEVICES WITH DIFFERENT DIMENSIONS AND DENSITIES

In this section we review all our results for the trivial and topological devices, in small ($(L_x, L_y) = (12, 6)$) and large ($(L_x, L_y) = (13, 14)$, main text) devices (see Supplementary Fig. 4 a-d) and for low and high density of active particles (see Methods, Experimental Setup).

H. Topological chiral edge motion

We initiate each device with particles on the top left corner and compare our experimental data with our model in Supplementary Fig. 4 e. In order to decrease the statistical uncertainty, we use the symmetry between opposite corners to include trajectories from the bottom right corner in the figures.

At long times, fewer particles contribute to the average because some have shorter trajectories than others. The agreement between model and theory is generally better for shorter times because the experimental average then includes more particles, from 150 to 300 particles.

I. Non-Hermitian topology : corner localization

We initiate each device with a uniform density of particles on every cell. The time evolution of the density density resembles that in Figs. 4 a,b of the main text, drawn for the large device with a large density of active particles, resulting in an accumulation of active particles at the corners. This increase in the density of particles at the corners is compared quantitatively using the Shannon entropy, in Supplementary Fig. 4g.

Because the entropy is sensitive to the noise in the density of particles, we reduce it by averaging the density over adjacent cells. The range for this averaging is estimated by a Fourier transform, to determine how much the distribution of particles

spreads away from uniformity. We obtain the spread Δk from which we deduce the range of fluctuations $\Delta r = 1/(2\Delta k)$. We then perform the averaging procedure over $3\Delta r \approx 3$ cells for the small devices, and ≈ 2 cells for the large devices. We perform this averaging procedure on both experimental and theoretical data.

The entropy we compute from our model is usually larger than what is observed experimentally. This can be caused by the residual noise from the fluctuations in the number of particles or by correlations between particles, that tend obstruct the motion of each other and lead to jamming. Also we see a difference between the entropies of the topological and trivial devices when the density of active particles is large. In this case the larger number of particles per cell enables the use of the entropy as a probe of the non-Hermitian skin effect.

SUPPLEMENTARY REFERENCES

[1] C. L. Kane, Ranjan Mukhopadhyay, and T. C. Lubensky, "Fractional quantum hall effect in an array of quantum wires," Phys. Rev. Lett. **88**, 036401 (2002).

[2] Tobias Meng, "Coupled-wire constructions: a Luttinger liquid approach to topology," European Physical Journal Special Topics **229**, 527–543 (2020).

[3] Thomas Iadecola, Titus Neupert, Claudio Chamon, and Christopher Mudry, "Wire constructions of abelian topological phases in three or more dimensions," Phys. Rev. B **93**, 195136 (2016).

[4] Kinjal Dasbiswas, Kranthi K. Mandadapu, and Suriyanarayanan Vaikuntanathan, "Topological localization in out-of-equilibrium dissipative systems," Proceedings of the National Academy of Sciences **115**, E9031–E9040 (2018).

[5] Kohei Kawabata, Ken Shiozaki, Masahito Ueda, and Masatoshi Sato, "Symmetry and topology in non-hermitian physics," Phys. Rev. X **9**, 041015 (2019).

[6] Kohei Kawabata, Masatoshi Sato, and Ken Shiozaki, "Higher-order non-hermitian skin effect," Phys. Rev. B **102**, 205118 (2020).

[7] W. P. Su, J. R. Schrieffer, and A. J. Heeger, "Solitons in polyacetylene," Phys. Rev. Lett. **42**, 1698–1701 (1979).

[8] Ching-Kai Chiu, Jeffrey C. Y. Teo, Andreas P. Schnyder, and Shinsei Ryu, "Classification of topological quantum matter with symmetries," Rev. Mod. Phys. **88**, 035005 (2016).

[9] Ananya Ghatak and Tanmoy Das, "New topological invariants in non-hermitian systems," Journal of Physics: Condensed Matter **31**, 263001 (2019).

[10] Nobuyuki Okuma, Kohei Kawabata, Ken Shiozaki, and Masatoshi Sato, "Topological origin of non-hermitian skin effects," Phys. Rev. Lett. **124**, 086801 (2020).

[11] Ching Hua Lee, Linhu Li, and Jiangbin Gong, "Hybrid higher-order skin-topological modes in nonreciprocal systems," Phys. Rev. Lett. **123**, 016805 (2019).

[12] Yuhao Ma and Taylor L. Hughes, "The Quantum Skin Hall Effect," arXiv e-prints , arXiv:2008.02284 (2020), arXiv:2008.02284 [cond-mat.mes-hall].

[13] Ryo Okugawa, Ryo Takahashi, and Kazuki Yokomizo, "Second-order topological non-Hermitian skin effects," arXiv e-prints , arXiv:2008.03721 (2020), arXiv:2008.03721 [cond-mat.mes-hall].

[14] Yongxu Fu and Shaolong Wan, "Non-Hermitian Second-Order Skin and Topological Modes," arXiv e-prints , arXiv:2008.09033 (2020), arXiv:2008.09033 [cond-mat.mes-hall].

[15] Frank Schindler, Zhijun Wang, Maia G Vergniory, Ashley M Cook, Anil Murani, Shamashis Sengupta, Alik Yu Kasumov, Richard Deblock, Sangjun Jeon, Ilya Drozdov, et al., "Higher-order topology in bismuth," Nature physics **14**, 918–924 (2018).
